# Pancytopenia With Subsequent Diagnosis of Hemophagocytic Lymphohistiocytosis in a Middle-Aged Male

**DOI:** 10.1155/crom/5526211

**Published:** 2025-10-31

**Authors:** Pavel Bleik, Steve Nwokeocha, Thanmay Sathi, Egor Zakharchenko, Day Hills

**Affiliations:** ^1^Department of Internal Medicine, Bassett Medical Center, Cooperstown, New York, USA; ^2^Department of Hematology and Oncology, Bassett Medical Center, Cooperstown, New York, USA

**Keywords:** hematology, HLH, pancytopenia

## Abstract

Hemophagocytic lymphohistiocytosis (HLH) is a rare, life-threatening hyperinflammatory syndrome resulting from uncontrolled activation of the immune system. It is characterized by persistent fever, cytopenias, organomegaly, and a constellation of laboratory abnormalities including hyperferritinemia, hypertriglyceridemia, hypofibrinogenemia, and elevated soluble interleukin-2 receptor levels. HLH can be broadly classified into primary (familial) and secondary forms, the latter often triggered by infections, malignancies, autoimmune diseases, or other systemic insults. Despite advancements in diagnostic criteria and therapeutic strategies, HLH continues to carry high morbidity and mortality, largely due to its nonspecific and variable presentation that often leads to delays in diagnosis. Early recognition and prompt initiation of immunosuppressive therapy are crucial to improving outcomes. We are presenting a case of symptomatically gastrointestinal-dominant HLH presentation potentially due to an unusual trigger of TMP/SMX in an otherwise healthy adult male who presented with flu-like symptoms accompanied with abdominal pain.

## 1. Case Presentation

A 52-year-old male with medical history significant only for mild hypertension, chronic persistent lower extremity rash for which he had been treated multiple times in the recent past with steroids and antibiotics, and prediabetes presented to a hospital with a chief complaint of nausea, vomiting, and abdominal pain going on for 3–4 days after he had been recently seen by a primary care physician in the settings of flu-like symptoms accompanied by nausea and generalized rash and got prescribed short course of trimethoprim/sulfamethoxazole (TMP/SMX). As he described, the first symptom was fever up to 101 F, then shortly thereafter, nausea accompanied by 1–2 episodes of vomiting on the first day. On the next day, abdominal pain developed around umbilical region which he had described as shooting, nonradiating, and 5/10 intensity. Lastly, he started to have nonbloody watery diarrhea.

Upon initial presentation, he denied any other complaints. The patient's BP was 113/68, pulse is 118, temperature is 37.6°C, and RR is 25.

His bloodwork upon presentation on a Day 1 was as follows: CBC was significant for WBC of 0.8 × 10e3 cells/*μ*L (normal range 3.7–10.8 × 10e3 cells/*μ*L), mild anemia with hemoglobin level of 10.9 g/dL (normal range 11.5–18.0 g/dL), MCV of 79.3 fL (normal range 81–99 fL), and platelet count of 86 × 10e3 cells/*μ*L (normal range 140–425 × 10e3 cells/*μ*L), along with absolute neutrophil count (ANC) was 500 cells/*μ*L (normal range 1500–7400 cells/*μ*L). Automatic differential did not reveal any blasts. Complete metabolic panel (CMP) showed sodium level of 126 mmol/L (normal range 136–145 mmol/L), calcium level of 7.2 mg/dL (normal range 8.6–10.3 mg/dL), and albumin of 2.3 g/dL (normal range 3.5–5.7 g/dL) as well as elevated AST of 109 U/L (normal range 13–39 U/L) and mildly elevated bilirubin of 1.4 mg/dL (normal range 0.3–1.0 mg/dL). Lactic acid was 4.8 mmol/L (reference range 0.5–2.2 mmol/L). The patient was diagnosed with sepsis by SIRS criteria, given IV fluids, and loading doses of vancomycin and Zosyn. He was transferred to our facility the same day (Day 1).

**Figure 1:**
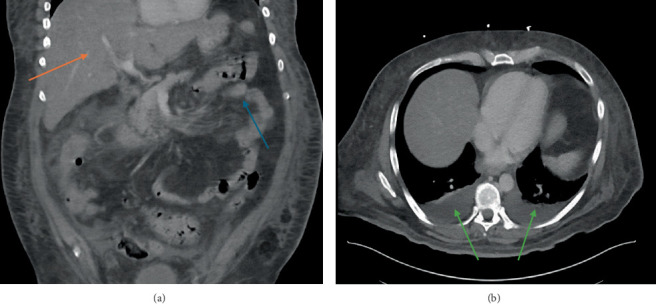
(a, b) CT scan showing splenic flexure colonic wall edema (blue arrow) along with fatty infiltration of the liver (red arrow). Bilateral pleural effusions (green arrows).

Upon presenting to our facility, his vital signs were as follows: BP 113/70, HR 111, temperature 99.6 F, and SpO2 = 96 on room air. Additional labs showed the following: LDH 1753 U/L (reference range 140–271 U/L), ferritin 39,145 ng/mL (reference range 16–243 ng/mL), triglycerides 583 mg/dL (reference range < 150 mg/dL), CRP 11.5 mg/dL (reference range 0.1–1.0 mg/dL), D-dimer 6251 ng/mL (reference range 215–499 ng/mL), and fibrinogen level 48 mg/dL (reference range 164–522 mg/dL). INR and PTT were also elevated at 1.8 and 41.4, respectively. CT scan of the abdomen and pelvis with IV contrast demonstrated thickening of the ascending, transverse, and splenic flexure of the colon, a moderate amount of fluid was found within the peritoneum, and a significant fatty infiltration of the liver was also observed (Figure 1a), along with moderate pleural effusions (Figure 1b).

Upon further questioning, the patient reported that he was relatively recently treated with Bactrim for left lower extremity cellulitis that had been an issue for the last couple of years. He has been having left lower extremity rash below the kneecap that would come and go. His treatment initially included short courses of antibiotics without significant success, and at some point, he started to get treated with short courses of prednisone with a good response. Occasionally, the flare-ups would demonstrate a clinical picture of cellulitis, and due to this, he would still occasionally get prescriptions of antibiotics (mostly doxycycline and Bactrim).

Vancomycin and Zosyn were started empirically upon admission, and the patient was also empirically covered with fluconazole due to lack of blood culture results at that time and also uncertainty and variety of clinical symptoms. Tick-borne panel, serum osmolality, and an additional set of bloodwork were collected. Surgery was evaluated, but the clinical and imaging findings were not consistent with a surgical abdomen, and no specific surgical recommendations were received.

On Day 2, an infectious disease specialist was consulted. They proposed a broad differential diagnosis including tick-borne disease, cellulitis, possible fungal-related sepsis, and infectious diarrhea and recommended empiric treatment for anaplasmosis pending immunohistochemical and molecular tick-borne panel results. Doxycycline therapy was initiated, and fluconazole was changed to caspofungin for broader coverage. The hematology service was consulted due to suspected bone marrow failure, and WBC, PLT, and RBC continued to drop.

Based on refractory fever, rapidly progressive pancytopenia, markedly elevated ferritin of > 4500, D-dimer elevation, LDH, and hypofibrinogenemia, HLH was raised as a diagnostic possibility in addition to acute myeloid leukemia or aggressive lymphoproliferative malignancy.

**Figure 2:**
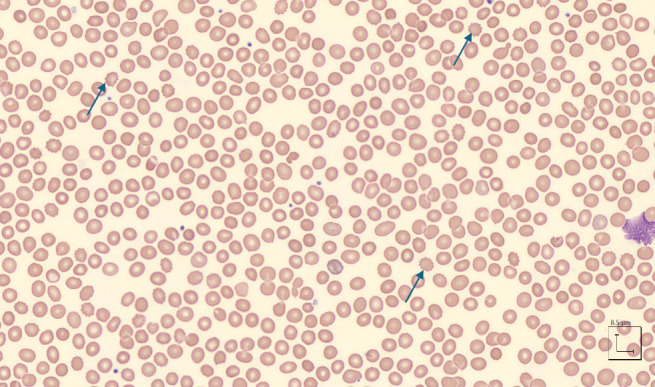
Blood smear showing no significant abnormalities except for a few elliptocytes (blue arrows).

Initial hematologic SPEP, UPEP, and peripheral blood flow cytometry were all normal or unremarkable. Peripheral blood smear did not reveal intracellular pathogens, blasts, or cellular monotonies other than prevalent Pelger–Huët cells, scant normal appearing thrombocytes, and reactive monocytes (Figure 2). Rapid fungal test and cryoglobulin test both were noncontributory.

**Figure 3:**
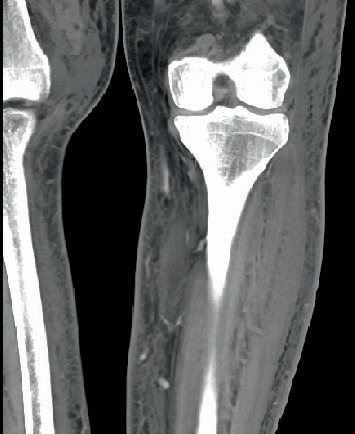
CT scan of the left lower extremity showing soft tissue swelling (blue arrows).

Stool studies were positive for enteroaggregative *Escherichia coli*, and antibiotic management was not changed as it was advised by Infectious disease specialist. CT of the left lower extremity was performed, due to persistent LLE swelling; this showed significant but nonspecific soft tissue inflammation without evidence of tumor or localized fluid collection (Figure 3).

Bone marrow biopsy was performed on Hospital Day 3. Shortly after the procedure, he developed bleeding at the biopsy site, requiring continuous pressure. He was transferred to the intensive care unit due to persistent bleeding and received fresh frozen plasma and platelet transfusions before bleeding abated.

While waiting for the bone marrow results, the patient was started on dexamethasone 10 mg twice daily.

**Figure 4:**
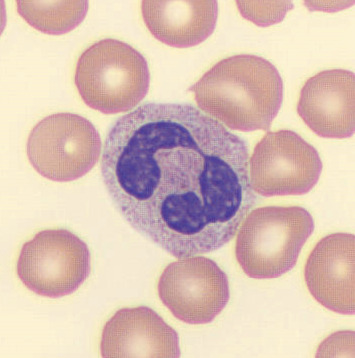
White blood cell demonstrating the Pelger–Huët sign (blue arrow).

Patient's platelet count continued to drop despite steroid therapy, along with WBC, and reached 23 × 10e3 and 0.3 × 10e3 cells/*μ*L, respectively, and platelet transfusion was given, resulting in an inappropriate response, with platelet count being within 26–19 × 10e3 cells/*μ*L after transfusion and, unexpectedly, continued to drop. Blood smear was repeated, and again, no significant abnormality was found except the Pelger–Huët sign, which is a nonspecific finding but can be seen in MDS or AML (Figure 4).

By Hospital Days 3–4, ferritin reached, reached 59316 U/L, triglycerides 683 mg/dL, CRP remained elevated, and severe pancytopenia persisted; his clinical HLH score was 236 (98%–99% probability of HLH). He fulfilled HLH-2004 criteria by the following: presence of high fever, cytopenia affecting two or more cell lines (all cell lines in this case), hypertriglyceridemia, serum ferritin concentration more than 500 ng/mL, and hepatosplenomegaly (the spleen in length was reported as 14 cm, and liver craniocaudal length was reported as 17 cm), overall already having five out of eight criteria of HLH-2004.

**Figure 5:**
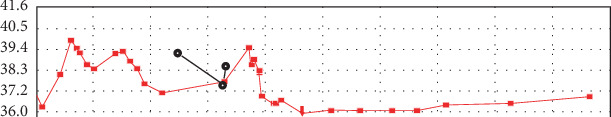
Fever curve during the admission. Each square represents 8 h interval. The blue arrow shows the timeline of steroid introduction.

In the ICU, he was continued on high-dose steroids with blood product support; though platelets continued to remain low and ferritin high, his fever curve began to drop (Figure 5).

**Figure 6:**
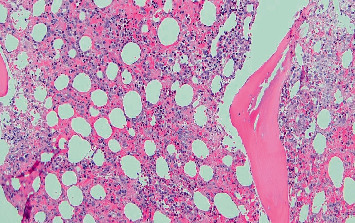
Bone marrow biopsy, 20×.

**Figure 7:**
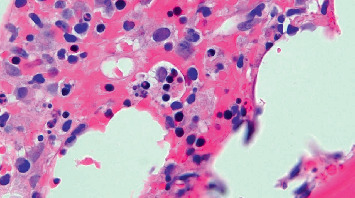
Bone marrow biopsy (×100) demonstrating erythrohistiocytosis (arrow).

Bone marrow biopsy showed cellular marrow with maturing hematopoiesis, hematophagocytosis, and histiocytic inflammation without an increased amount of blast; overall findings considered consistent with hemophagocytic lymphohistiocytosis (Figures 6 and 7 and Supporting Information).

Remarkably, soluble IL-2R which had been sent earlier, returned high—12,365 (normal range 158–623 U/mL), and the chemokine ligand was 322,439.

On Hospital Day 4, the patient was transferred to a higher level of care and the potential initiation of HLH chemotherapy protocol. At this facility, additional testing for potential underlying/triggering infection was performed. *Francisella tularensis*; HIV; hepatitides A, B, and C; *Clostridium difficile*; *Histoplasma*; *Legionella*; *Leptospira*; *Leishmania*; human herpes virus; parvovirus; and respiratory viral panel along with EBV and CMV were all negative, except for *Chlamydia psittaci* IgG. Tick panel returned negative. Antibiotic regimen was modified to include doxycycline, atovaquone, and ciprofloxacin as a broad and empiric coverage for *Chlamydia psitacci* and neutropenic fever.

Additionally, the autoimmune panel, including rheumatoid factor, P-ANCA, C-ANCA, ANA, anti-ds-DNA, anti-scl-70, anticentromere, anti-Ro and anti-La, anti-CCP, and antihistone-ab, was negative. Free light chain ratio was 0.74.

Due to a chronic lower extremity rash, a biopsy was performed to rule out cutaneous lymphoma (which could be a trigger for malignancy-associated HLH), but this showed nonspecific findings of occasional focal dermal hemorrhage and minimal inflammation.

HLH94 protocol with etoposide and dexamethasone was initiated on Hospital Day 5. BSA was calculated as 2.26 m^2^. HLH94 includes 150 mg/m^2^ IV of etoposide twice a week for 2 weeks and 150 mg/m^2^ IV weekly till the end of the week; 8.10 days after starting the treatment, the patient's fever and diarrhea resolved; however, the WBC count remained at 0.1 and remained transfusion dependent because of profound thrombocytopenia.

While completing HLH94 and being on broad-spectrum antibiotics, given the unremarkable workup for any secondary causes but remarkable fever up to 101.4, the patient was started on filgrastim on Day 11 after initiation of HLH-94, and a steroid taper was initiated earlier than planned. Two days after being on filgrastim, the patient's white count has improved and reached 17 × 10e3 cells/*μ*L (ref. range 3.5–11.5 × 10e3 cells/*μ*L). His platelets also reached 167, and his hemoglobin level remained stable. He was noted to have a marked improvement in ferritin level, and at the time of discharge, it reached 4780 ng/mL. Subsequently, a week later, the patient experienced a fulminant decline accompanied by abdominal pain, blood pressure drop, and hypothermia. He was diagnosed with peritonitis due to organ perforation and was taken straight to the OR. Abdominal washout was performed right away. Unfortunately, due to uncontrolled sepsis and multiorgan failure, the patient passed away shortly after the procedure. The latest blood cultures were positive for *Enterococcus faecium* along with positive beta-D glucan.

## 2. Discussion

HLH is divided into primary or familial (FHLH) and secondary or acquired types. FHLH is typically diagnosed in infants or young children, for which hematopoietic stem cell transplantation (HSCT) is currently the potentially definitive treatment [1]. Identification of an implicated gene in a patient with clinical HLH helps confirm the diagnosis [2]. A few variants have milder clinical courses and can have adult-onset, usually requiring environmental triggers [2, 3]. Mutations in genes involving T-lymphocyte regulation dominate familial HLH genetic findings [4–6].

The etiology of secondary HLH is usually an immune hyperinflammatory/dysregulatory response to infection (usually viral, less often nonviral), or inflammation, including malignancy-associated inflammation, rheumatologic disorders, or, more recently, iatrogenic inflammation secondary to CAR-T cell therapy [1, 7, 8]. A few reports suggest that medication can induce HLH, including TMP/SMX, which our patient was exposed to prior to hospitalization, as well as immune checkpoint inhibitors [9–11].

Both primary and secondary HLH have similar clinical and laboratory findings: persistent fever, abdominal pain, organomegaly especially hepatosplenomegaly, CNS manifestations including isolated CNS HLH and encephalopathy, rashes such as petechiae and purpuras, pancytopenia, significant ferritin and triglyceride elevations, low fibrinogen, elevated soluble interleukin-2 receptor alpha (CD-25), and low natural killer (NK) cell activity [1, 10, 12, 13]. As with our patient, patients typically present with a sepsis-like picture, and diagnosis is difficult in critically ill patients who would be thought to have severe sepsis with multiorgan dysfunction or a vague tick-borne illness. Severe gastrointestinal symptoms are not frequently reported; in our patient, it was initially thought that he had severe sepsis secondary to colitis.

The HLH-2024 criteria were developed based on a case control study to validate the HLH-2004 criteria which itself was based mainly on expert opinion in mostly pediatric patients from the HLH-94 study, eventually showing that the clinical variables, except for low/absent NK activity, have significant validity in clinical practice in the diagnosis of FHLH [14, 15]. The criteria are also useful in diagnosing reactive HLH, differentiating it from infection, and can be used alongside the HLH probability score (Hscore) which helps determine the probability of a diagnosis of HLH in a patient, with higher scores signifying higher probabilities [16]. The OHI index was also built on the work of HLH-2004 to create predictive diagnostic criteria for HLH in malignancies [14, 17–19]. Some authors have also argued that the HLH-2004 criteria do not include features “almost always” present such as liver inflammation and relatively common neurological findings [5].

To consolidate a diagnosis of HLH, a bone marrow biopsy can be performed which would typically show the characteristic features of hemophagocytosis (although not regarded by Jordan et al. as sensitive or specific enough to be a major diagnostic criteria), and genetic testing can be considered in young patients, or those with recurring illness, for numerous autosomal recessive genes and X-linked lymphoproliferative syndromes [4–6, 20].

Familial HLH is typically diagnosed in children under the age of 1, with reported in utero cases, but can be diagnosed at any age even in adults, while secondary HLH is typically diagnosed in older children and adults [3, 5, 21].

The incidence of HLH diagnosis is rising [8, 22]. In England in 2018, it was estimated at 4.2 cases per million population, with rates increasing fourfold between 2003 and 2018 [8]. Among adults admitted in the United States, there were 16,136 HLH-related admissions between 2006 and 2019, with incidence increasing yearly, and higher in-hospital mortality rates among older patients and patients whose HLH was associated with congenital immunodeficiency syndrome, malignancies, and infection, in that order [22]. The increasing incidence rates of diagnosis and admissions may be related in part to increasing awareness, diagnostic criteria, testing (e.g., soluble IL-2 and genetic mutation), and increasing knowledge of a rare and still underdiagnosed condition [22]. Additionally, the increases in incidence of malignancy and rheumatologic diagnoses such as IBD and related use of immune and T cell therapies may also contribute [8, 22].

Treatment of HLH is usually needed, irrespective of underlying triggers and their management, because once established, the HLH-associated hyperinflammation persists without treatment [3]. In some cases, a wait-and-watch approach may be taken in clinically stable patients whose HLH parameters are improving with underlying disease-specific treatment [23].

While certain cases may achieve remission with only high-dose steroids with very slow taper [10], HLH-94 remains the standard treatment for both primary and secondary HLH, and treatment of adult cases needs individualized tailoring [23, 24]. FHLH tends to recur and usually requires HSCT for a potentially curative effect [23, 25]. Refractory cases are managed based on the underlying trigger and patient factors, with a range of therapeutic options [23, 26].

The HLH-94 protocol entails an initial period of therapy for 8 weeks which aims at achieving clinical remission [23, 24, 27]. This consists of etoposide 150 mg/m^2^ twice weekly for 2 weeks and then weekly for 6 weeks and dexamethasone which is initially given at high dose for 2 weeks and gradually tapered [23, 27]. After this point, patients with known FHLH or persistent nonfamilial HLH should receive continuation therapy, which includes dexamethasone 10 mg/kg^2^ for 3 days every 2nd week and etoposide infusions every alternate 2nd week in combination with daily oral cyclosporine. The objective of the continuation therapy is to keep patients in a stable condition during the search for a marrow donor [23, 27]. The study highlighted that the 3-year survival for patients who underwent HSCT was 62%, and 20% of patients achieved long-term remission donor [27].

The HLH-2004 protocol was planned with an objective to provide immunosuppression from the beginning of treatment, and the primary difference compared to the HLH-94 protocol is the introduction of cyclosporine A from the beginning [17]. Further studies showed that the HLH-2004 protocol resulted in a slightly higher pre-HSCT transplantation survival rate (81% vs. 73% in HLH-94), but the overall 5-year survival rates were not significantly different (59% vs. 50%) [15].

In addition to HLH-94, other treatments are increasingly in use in the treatment of HLH. Emapalumab is a human anti-interferon gamma antibody and has emerged as a promising treatment for HLH, especially in patients with refractory, recurrent, or progressive disease [28]. Many clinical trials have demonstrated the ability of ruxolitinib, a JAK inhibitor, to suppress inflammatory cytokine production and improve clinical outcomes in HLH [29]. Alemtuzumab is a monoclonal antibody targeting CD52, and studies reported high pretransplantation survival rates among previously untreated patients [30]. There are other therapies under investigation such as anakinra, tocilizumab, and anti-IL-18 therapies [31].

We judged our patient to have probable infection-associated HLH; however, due to TMP/SMX exposure just prior to presentation, it also raised the suspicion that it was a potential trigger which we aimed to highlight by writing this case. His gastrointestinal-dominant presentation was also worth noting, especially with his clinical outcome of recurrent perforation and development of intra-abdominal collections of possible bleeding and/or pus which could not be ascertained due to his quick progression to eventual death before they could be drained. Finally, although he received aggressive treatment, his dismal outcome appears to have occurred due to his already immunosuppressed and pancytopenic state secondary to the treatment he received, highlighting further the challenges of HLH management.

## 3. Conclusion

This case demonstrates the potentially confusing clinical presentation of a hyperinflammatory state which is the hallmark of HLH (in this particular case, gastrointestinal form) and can be missed due to its similarities with critical illnesses such as severe sepsis, pointing out the need to have a high index of suspicion for this entity in the appropriate clinical setting, including within patients presenting with gastrointestinal symptoms. It also highlights further unusual triggers and presentations of the disorder.

## Data Availability

The data supporting the findings of this case report are available from the corresponding author upon reasonable request. All relevant clinical and laboratory data are included within the manuscript and its supporting information. Due to patient confidentiality and privacy concerns, patient-specific data cannot be shared publicly.

## Nomenclature

HLH: hemophagocytic lymphohistiocytosis
BP: blood pressure
RR: respiratory rate
CBC: complete blood count
WBC: white blood cells
MCV: mean corpuscular volume
ANC: absolute neutrophil count
CMP: complete metabolic panel
SIRS: systemic inflammatory response syndrome
LDH: lactate dehydrogenase
CRP: C-reactive protein
INR: international normalized ratio
PTT: partial thromboplastin time
CT: computed tomography
IV: intravenous
SPEP: serum protein electrophoresis
UPEP: urine protein electrophoresis
MDS: myelodysplastic syndrome
AML: acute myeloid leukemia
ICU: intensive care unit
CD: cluster of differentiation
IgG: immunoglobulin G
RF: rheumatoid factor
P/C-ANCA: P/C-antineutrophilic cytoplasmic antibodies
ANA: antinuclear antibodies
Anti-ds-DNA: antidouble-stranded DNA
Anti-scl: antiscleroderma antibodies
Anti-CCP: anticyclic citrullinated peptide
Ab: antibodies
BSA: body surface area

## Supporting Information

Supporting Information Additional supporting information can be found online in the Supporting Information section. Bone marrow biopsy description: Cellular marrow with maturing hematopoiesis, hemophagocytosis, and histiocytic inflammation, without monotypic B-cell or aberrant T-cell population. Blasts are not increased.
