# Supplementary material for: Pancytopenia With Subsequent Diagnosis of Hemophagocytic Lymphohistiocytosis in a Middle-Aged Male
Source: Case Rep Oncol Med. 2025 Oct 31;2025:5526211. doi: 10.1155/crom/5526211 (PMC12595237; doi:10.1155/crom/5526211)
Supplement: Supporting Information — Additional supporting information can be found online in the Supporting Information section. Bone marrow biopsy description: Cellular marrow with maturing hematopoiesis, hemophagocytosis, and histiocytic inflammation, without monotypic B-cell or aberrant T-cell population. Blasts are not increased. [file 5526211.f1.docx]

Bone marrow biopsy description:

Aspirate flow cytometry revealed the lymphocyte gate content 45% of total events of which 9% are CD19/CD20 positive B-cells with a kappa/lambda ratio of 1.8, 82% are CD3-positive T-cells with a CD4/CD8 ratio of 6.7, and 9% are CD3 negative /CD7 positive NK cells. The CD45 (dim) low side-scatter blast gate contains 10% of total events; CD34 positive blasts are <1% of total events. Granulocytes demonstrate aberrant expression of CD64. Subsequent hemopathology report revealed numerous CD 163/CD 68 positive macrophages and histiocytes present within the marrow space with occasional aggregates around normal mononuclear cells. Numerous CD3+ T-cells and CD20+- B Cells were decreased.
